# 4-Chloro-*N*-(2-chloro­benzo­yl)benzene­sulfonamide

**DOI:** 10.1107/S1600536810019057

**Published:** 2010-05-26

**Authors:** B. Thimme Gowda, Sabine Foro, P. A. Suchetan, Hartmut Fuess

**Affiliations:** a Department of Chemistry, Mangalore University, Mangalagangotri 574 199, Mangalore, India; b Institute of Materials Science, Darmstadt University of Technology, Petersenstrasse 23, D-64287 Darmstadt, Germany

## Abstract

In the structure of the title compound, C_13_H_9_Cl_2_NO_3_S, the conformation of the N—H bond in the C—SO_2_—NH—C(O) segment is *anti* to the C=O bond. The mol­ecule is twisted at the S atom with a torsion angle of 65.7 (2)°. The dihedral angle between the sulfonyl benzene ring and the —SO_2_—NH—C—O segment is 88.5 (1)°, and that between the sulfonyl and the benzoyl benzene rings is 58.0 (1)°. In the crystal, mol­ecules are linked by pairs of N—H⋯O hydrogen bonds, forming inversion dimers.

## Related literature

For our study of the effect of ring and side-chain substituents on the crystal structures of *N*-aromatic sulfonamides and for related structures, see: Gowda *et al.* (2010 ▶); Suchetan *et al.* (2010**a* ▶,*b* ▶,c* ▶).
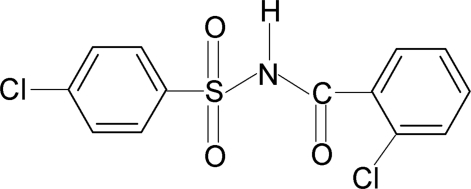


## Experimental

### 

#### Crystal data

C_13_H_9_Cl_2_NO_3_S
*M* *_r_* = 330.17
Triclinic, 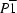

*a* = 6.3882 (9) Å
*b* = 10.311 (1) Å
*c* = 11.171 (1) Å
α = 79.01 (1)°
β = 74.47 (1)°
γ = 84.76 (1)°
*V* = 695.32 (13) Å^3^
*Z* = 2
Mo *K*α radiation
μ = 0.62 mm^−1^
*T* = 299 K
0.24 × 0.20 × 0.14 mm

#### Data collection

Oxford Diffraction Xcalibur diffractometer with a Sapphire CCD detector
Absorption correction: multi-scan (*CrysAlis RED*; Oxford Diffraction, 2009 ▶) *T*_min_ = 0.865, *T*_max_ = 0.918
4417 measured reflections
2825 independent reflections
2378 reflections with *I* > 2σ(*I*)
*R*_int_ = 0.016

#### Refinement

*R*[*F* ^2^ > 2σ(*F* ^2^)] = 0.039
*wR*(*F* ^2^) = 0.107
*S* = 1.06
2825 reflections
184 parameters
1 restraint
H atoms treated by a mixture of independent and constrained refinement
Δρ_max_ = 0.25 e Å^−3^
Δρ_min_ = −0.52 e Å^−3^

### 

Data collection: *CrysAlis CCD* (Oxford Diffraction, 2009 ▶); cell refinement: *CrysAlis RED* (Oxford Diffraction, 2009 ▶); data reduction: *CrysAlis RED*; program(s) used to solve structure: *SHELXS97* (Sheldrick, 2008 ▶); program(s) used to refine structure: *SHELXL97* (Sheldrick, 2008 ▶); molecular graphics: *PLATON* (Spek, 2009 ▶); software used to prepare material for publication: *SHELXL97*.

## Supplementary Material

Crystal structure: contains datablocks I, global. DOI: 10.1107/S1600536810019057/xu2765sup1.cif

Structure factors: contains datablocks I. DOI: 10.1107/S1600536810019057/xu2765Isup2.hkl

Additional supplementary materials:  crystallographic information; 3D view; checkCIF report

## Figures and Tables

**Table 1:** Hydrogen-bond geometry (Å, °)

*D*—H⋯*A*	*D*—H	H⋯*A*	*D*⋯*A*	*D*—H⋯*A*
N1—H1*N*⋯O2^i^	0.81 (2)	2.13 (2)	2.914 (2)	164 (2)

Symmetry code: (i) 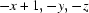.

**Fig. 1.**
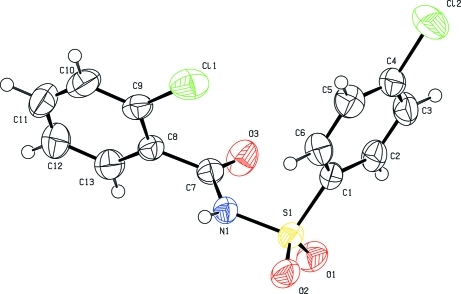
Molecular structure of the title compound, showing the atom-labelling scheme. Displacement ellipsoids are drawn at the 50% probability level.

**Fig. 2.**
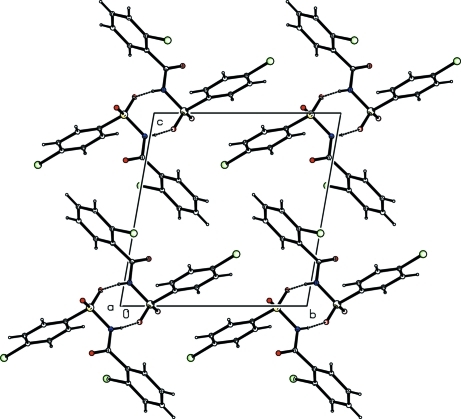
Molecular packing in the title compound. Hydrogen bonds are shown as dashed lines.

## supplementary crystallographic information

### Comment

As a part of studying the effect of ring and the side chain substituents on the crystal structures of *N*-aromatic sulfonamides (Gowda *et al.*, 2010; Suchetan *et al.*, 2010*a,b,c*), the structure of *N*-(2-chlorobenzoyl)-4-chlorobenzenesulfonamide (I) has been determined. The conformation of the N—H bond in the C—SO_2_—NH—C(O) segment is *anti* to the C=O bond (Fig. 1), similar to those observed in *N*-(2-chlorobenzoyl)-benzenesulfonamide (II) (Gowda *et al.*, 2010), *N*-(benzoyl)-4-chlorobenzenesulfonamide (III) (Suchetan *et al.*, 2010*a*), *N*-(2-chlorobenzoyl)-2-chlorobenzenesulfonamide (IV) (Suchetan *et al.*, 2010*b*) and *N*-(4-chlorobenzoyl)-4- chlorobenzenesulfonamide (V) (Suchetan *et al.*, 2010*c*).

Further, the conformation of the C=O bond in the C—SO_2_—NH—C(O) segment of (I) is *syn* to the *ortho*-Cl in the benzoyl ring, similar to that observed in (II) and (IV).

The molecules are twisted at the *S* atom with the torsional angle of 65.7 (2)°, compared to those of -59.0 (2)° (molecule 1) and -67.3 (2)° (molecule 2) in (II), -70.0 (2)°, 61.3 (2)° in the two independent molecules of (III), 66.5 (2)° in (IV) and 67.5 (3)° in (V).

The dihedral angle between the sulfonyl benzene ring and the —SO_2_—NH—C—O segment is 88.5 (1)°, compared to the values of 87.3 (1)° (molecule 1) and 73.3 (1)° (molecule 2) in (II), 72.0 (1)° & 77.3 (1)° in the two molecules of (III), 86.9 (1)° in (IV) and 79.0 (1)° in (V).

Furthermore, the dihedral angle between the sulfonyl and the benzoyl benzene rings is 58.0 (1)°, compared to the values of 69.8 (1)° (molecule 1) and 89.8 (1)° (molecule 2) in (II), 62.8 (1)° (molecule 1) and 78.6 (1)° (molecule 2) of (III), 76.9 (1)° in (IV) and 85.6 (1)° in (V)

The packing of molecules linked by of N—H···O(S) hydrogen bonds (Table 1) is shown in Fig. 2.

### Experimental

The title compound was prepared by refluxing a mixture of 2-chlorobenzoic acid, 4-chlorobenzenesulfonamide and phosphorous oxy chloride for 3 h on a water bath. The resultant mixture was cooled and poured into ice cold water. The solid obtained was filtered, washed thoroughly with water and then dissolved in sodium bicarbonate solution. The compound was later reprecipitated by acidifying the filtered solution with dilute HCl. It was filtered, dried and recrystallized.

Prism like colourless single crystals of the title compound used in X-ray diffraction studies were obtained by slow evaporation of its toluene solution at room temperature.

### Refinement

The H atoms of the NH groups were located in a difference map and later restrained to N—H = 0.86 (2) %A. The other H atoms were positioned with idealized geometry using a riding model with C—H = 0.93 Å. All H atoms were refined with isotropic displacement parameters (set to 1.2 times of the *U*_eq_ of the parent atom).

### Crystal data

**Table:**

C_13_H_9_Cl_2_NO_3_S	*Z* = 2
*M**_r_* = 330.17	*F*(000) = 336
Triclinic, *P*1	*D*_x_ = 1.577 Mg m^−^^3^
Hall symbol: -P 1	Mo *K*α radiation, λ = 0.71073 Å
*a* = 6.3882 (9) Å	Cell parameters from 2339 reflections
*b* = 10.311 (1) Å	θ = 2.5–27.8°
*c* = 11.171 (1) Å	µ = 0.62 mm^−^^1^
α = 79.01 (1)°	*T* = 299 K
β = 74.47 (1)°	Prism, colourless
γ = 84.76 (1)°	0.24 × 0.20 × 0.14 mm
*V* = 695.32 (13) Å^3^	

### Data collection

**Table:**

Oxford Diffraction Xcalibur diffractometer with a Sapphire CCD detector	2825 independent reflections
Radiation source: fine-focus sealed tube	2378 reflections with *I* > 2σ(*I*)
graphite	*R*_int_ = 0.016
ω and φ scans	θ_max_ = 26.4°, θ_min_ = 2.5°
Absorption correction: multi-scan (*CrysAlis RED*; Oxford Diffraction, 2009)	*h* = −7→7
*T*_min_ = 0.865, *T*_max_ = 0.918	*k* = −12→11
4417 measured reflections	*l* = −13→13

### Refinement

**Table:**

Refinement on *F*^2^	Primary atom site location: structure-invariant direct methods
Least-squares matrix: full	Secondary atom site location: difference Fourier map
*R*[*F*^2^ > 2σ(*F*^2^)] = 0.039	Hydrogen site location: inferred from neighbouring sites
*wR*(*F*^2^) = 0.107	H atoms treated by a mixture of independent and constrained refinement
*S* = 1.06	*w* = 1/[σ^2^(*F*_o_^2^) + (0.0603*P*)^2^ + 0.1863*P*] where *P* = (*F*_o_^2^ + 2*F*_c_^2^)/3
2825 reflections	(Δ/σ)_max_ = 0.022
184 parameters	Δρ_max_ = 0.25 e Å^−^^3^
1 restraint	Δρ_min_ = −0.52 e Å^−^^3^

### Special details

**Table:**

Geometry. All esds (except the esd in the dihedral angle between two l.s. planes) are estimated using the full covariance matrix. The cell esds are taken into account individually in the estimation of esds in distances, angles and torsion angles; correlations between esds in cell parameters are only used when they are defined by crystal symmetry. An approximate (isotropic) treatment of cell esds is used for estimating esds involving l.s. planes.
Refinement. Refinement of *F*^2^ against ALL reflections. The weighted *R*-factor wR and goodness of fit *S* are based on *F*^2^, conventional *R*-factors *R* are based on *F*, with *F* set to zero for negative *F*^2^. The threshold expression of *F*^2^ > σ(*F*^2^) is used only for calculating *R*-factors(gt) etc. and is not relevant to the choice of reflections for refinement. *R*-factors based on *F*^2^ are statistically about twice as large as those based on *F*, and *R*- factors based on ALL data will be even larger.

### Fractional atomic coordinates and isotropic or equivalent isotropic displacement parameters (Å^2^)

**Table:**

	*x*	*y*	*z*	*U*_iso_*/*U*_eq_	
C1	0.3004 (3)	0.27841 (17)	0.07988 (17)	0.0364 (4)	
C2	0.1714 (3)	0.39202 (19)	0.0968 (2)	0.0464 (5)	
H2	0.0437	0.4059	0.0703	0.056*	
C3	0.2338 (4)	0.4844 (2)	0.1532 (2)	0.0561 (6)	
H3	0.1510	0.5624	0.1634	0.067*	
C4	0.4198 (4)	0.4596 (2)	0.1942 (2)	0.0520 (5)	
C5	0.5482 (4)	0.3465 (2)	0.1791 (2)	0.0543 (5)	
H5	0.6730	0.3317	0.2085	0.065*	
C6	0.4895 (3)	0.2553 (2)	0.1198 (2)	0.0478 (5)	
H6	0.5760	0.1791	0.1068	0.057*	
C7	0.0089 (3)	0.02215 (18)	0.22952 (18)	0.0384 (4)	
C8	0.0163 (3)	−0.09961 (18)	0.32606 (17)	0.0396 (4)	
C9	0.1632 (3)	−0.1155 (2)	0.39978 (18)	0.0451 (4)	
C10	0.1579 (5)	−0.2246 (3)	0.4941 (2)	0.0641 (7)	
H10	0.2565	−0.2347	0.5436	0.077*	
C11	0.0073 (6)	−0.3171 (3)	0.5141 (2)	0.0757 (8)	
H11	0.0043	−0.3908	0.5771	0.091*	
C12	−0.1402 (5)	−0.3025 (3)	0.4422 (3)	0.0730 (7)	
H12	−0.2424	−0.3663	0.4568	0.088*	
C13	−0.1370 (4)	−0.1940 (2)	0.3487 (2)	0.0554 (5)	
H13	−0.2378	−0.1840	0.3007	0.066*	
Cl1	0.34983 (10)	0.00381 (7)	0.37663 (6)	0.0664 (2)	
Cl2	0.49683 (16)	0.57327 (7)	0.26873 (8)	0.0938 (3)	
N1	0.1748 (3)	0.02815 (15)	0.12059 (15)	0.0398 (4)	
H1N	0.276 (3)	−0.0255 (19)	0.114 (2)	0.048*	
O1	0.0294 (3)	0.20637 (15)	−0.02823 (14)	0.0550 (4)	
O2	0.4074 (3)	0.11865 (14)	−0.08329 (13)	0.0537 (4)	
O3	−0.1307 (2)	0.10807 (16)	0.24443 (16)	0.0602 (4)	
S1	0.22277 (8)	0.15972 (4)	0.00846 (4)	0.04047 (15)	

### Atomic displacement parameters (Å^2^)

**Table:**

	*U*^11^	*U*^22^	*U*^33^	*U*^12^	*U*^13^	*U*^23^
C1	0.0364 (9)	0.0325 (8)	0.0360 (9)	−0.0001 (7)	−0.0058 (7)	−0.0008 (7)
C2	0.0448 (11)	0.0390 (10)	0.0503 (11)	0.0069 (8)	−0.0122 (9)	0.0006 (8)
C3	0.0695 (14)	0.0344 (10)	0.0568 (13)	0.0062 (9)	−0.0075 (11)	−0.0055 (9)
C4	0.0665 (14)	0.0424 (11)	0.0432 (11)	−0.0177 (9)	−0.0044 (10)	−0.0045 (8)
C5	0.0468 (11)	0.0561 (13)	0.0610 (13)	−0.0108 (9)	−0.0159 (10)	−0.0051 (10)
C6	0.0382 (10)	0.0431 (10)	0.0617 (13)	0.0040 (8)	−0.0140 (9)	−0.0091 (9)
C7	0.0365 (9)	0.0413 (9)	0.0406 (9)	−0.0023 (7)	−0.0146 (7)	−0.0076 (7)
C8	0.0430 (10)	0.0399 (9)	0.0342 (9)	0.0004 (7)	−0.0076 (7)	−0.0064 (7)
C9	0.0464 (10)	0.0529 (11)	0.0364 (10)	0.0068 (8)	−0.0111 (8)	−0.0120 (8)
C10	0.0786 (16)	0.0707 (15)	0.0399 (11)	0.0199 (13)	−0.0188 (11)	−0.0076 (10)
C11	0.112 (2)	0.0556 (14)	0.0457 (13)	0.0047 (14)	−0.0087 (14)	0.0059 (11)
C12	0.095 (2)	0.0539 (14)	0.0628 (16)	−0.0230 (13)	−0.0095 (14)	0.0015 (12)
C13	0.0615 (13)	0.0548 (12)	0.0500 (12)	−0.0148 (10)	−0.0127 (10)	−0.0059 (10)
Cl1	0.0571 (4)	0.0895 (4)	0.0633 (4)	−0.0126 (3)	−0.0273 (3)	−0.0178 (3)
Cl2	0.1363 (7)	0.0706 (5)	0.0834 (5)	−0.0386 (4)	−0.0236 (5)	−0.0259 (4)
N1	0.0441 (9)	0.0340 (8)	0.0394 (8)	0.0013 (6)	−0.0095 (7)	−0.0049 (6)
O1	0.0577 (9)	0.0586 (9)	0.0546 (9)	0.0005 (7)	−0.0303 (7)	−0.0030 (7)
O2	0.0656 (9)	0.0503 (8)	0.0367 (7)	0.0079 (7)	−0.0037 (6)	−0.0050 (6)
O3	0.0483 (8)	0.0617 (9)	0.0603 (10)	0.0152 (7)	−0.0092 (7)	−0.0008 (7)
S1	0.0464 (3)	0.0387 (3)	0.0356 (3)	0.00192 (18)	−0.01273 (19)	−0.00320 (18)

### Geometric parameters (Å, °)

**Table:**

C1—C6	1.382 (3)	C8—C13	1.385 (3)
C1—C2	1.383 (3)	C8—C9	1.386 (3)
C1—S1	1.7537 (19)	C9—C10	1.382 (3)
C2—C3	1.377 (3)	C9—Cl1	1.727 (2)
C2—H2	0.9300	C10—C11	1.363 (4)
C3—C4	1.370 (3)	C10—H10	0.9300
C3—H3	0.9300	C11—C12	1.375 (4)
C4—C5	1.374 (3)	C11—H11	0.9300
C4—Cl2	1.733 (2)	C12—C13	1.375 (3)
C5—C6	1.379 (3)	C12—H12	0.9300
C5—H5	0.9300	C13—H13	0.9300
C6—H6	0.9300	N1—S1	1.6510 (16)
C7—O3	1.200 (2)	N1—H1N	0.810 (15)
C7—N1	1.379 (3)	O1—S1	1.4185 (15)
C7—C8	1.497 (3)	O2—S1	1.4311 (15)
			
C6—C1—C2	121.20 (18)	C10—C9—C8	120.5 (2)
C6—C1—S1	118.92 (14)	C10—C9—Cl1	119.64 (18)
C2—C1—S1	119.87 (15)	C8—C9—Cl1	119.82 (16)
C3—C2—C1	119.35 (19)	C11—C10—C9	119.6 (2)
C3—C2—H2	120.3	C11—C10—H10	120.2
C1—C2—H2	120.3	C9—C10—H10	120.2
C4—C3—C2	119.03 (19)	C10—C11—C12	120.7 (2)
C4—C3—H3	120.5	C10—C11—H11	119.7
C2—C3—H3	120.5	C12—C11—H11	119.7
C3—C4—C5	122.2 (2)	C13—C12—C11	120.2 (2)
C3—C4—Cl2	119.51 (17)	C13—C12—H12	119.9
C5—C4—Cl2	118.31 (18)	C11—C12—H12	119.9
C4—C5—C6	119.0 (2)	C12—C13—C8	120.0 (2)
C4—C5—H5	120.5	C12—C13—H13	120.0
C6—C5—H5	120.5	C8—C13—H13	120.0
C5—C6—C1	119.17 (18)	C7—N1—S1	124.25 (13)
C5—C6—H6	120.4	C7—N1—H1N	121.9 (16)
C1—C6—H6	120.4	S1—N1—H1N	112.1 (16)
O3—C7—N1	122.05 (18)	O1—S1—O2	119.26 (9)
O3—C7—C8	123.12 (18)	O1—S1—N1	110.18 (9)
N1—C7—C8	114.82 (15)	O2—S1—N1	103.74 (8)
C13—C8—C9	119.07 (19)	O1—S1—C1	108.96 (9)
C13—C8—C7	119.35 (17)	O2—S1—C1	109.21 (9)
C9—C8—C7	121.40 (17)	N1—S1—C1	104.43 (8)
			
C6—C1—C2—C3	0.7 (3)	C8—C9—C10—C11	0.2 (3)
S1—C1—C2—C3	179.87 (16)	Cl1—C9—C10—C11	178.50 (19)
C1—C2—C3—C4	−1.7 (3)	C9—C10—C11—C12	−0.5 (4)
C2—C3—C4—C5	1.0 (3)	C10—C11—C12—C13	0.1 (4)
C2—C3—C4—Cl2	−178.50 (16)	C11—C12—C13—C8	0.6 (4)
C3—C4—C5—C6	0.6 (3)	C9—C8—C13—C12	−0.9 (3)
Cl2—C4—C5—C6	−179.85 (17)	C7—C8—C13—C12	−176.2 (2)
C4—C5—C6—C1	−1.6 (3)	O3—C7—N1—S1	9.1 (3)
C2—C1—C6—C5	0.9 (3)	C8—C7—N1—S1	−171.37 (13)
S1—C1—C6—C5	−178.23 (16)	C7—N1—S1—O1	−51.19 (18)
O3—C7—C8—C13	70.4 (3)	C7—N1—S1—O2	−179.94 (15)
N1—C7—C8—C13	−109.1 (2)	C7—N1—S1—C1	65.68 (17)
O3—C7—C8—C9	−104.8 (2)	C6—C1—S1—O1	−178.06 (15)
N1—C7—C8—C9	75.7 (2)	C2—C1—S1—O1	2.78 (18)
C13—C8—C9—C10	0.4 (3)	C6—C1—S1—O2	−46.22 (18)
C7—C8—C9—C10	175.66 (18)	C2—C1—S1—O2	134.61 (15)
C13—C8—C9—Cl1	−177.81 (16)	C6—C1—S1—N1	64.23 (17)
C7—C8—C9—Cl1	−2.6 (3)	C2—C1—S1—N1	−114.93 (16)

### Hydrogen-bond geometry (Å, °)

**Table:**

*D*—H···*A*	*D*—H	H···*A*	*D*···*A*	*D*—H···*A*
N1—H1N···O2^i^	0.81 (2)	2.13 (2)	2.914 (2)	164 (2)

Symmetry codes: (i) −*x*+1, −*y*, −*z*.

## References

[bb1] Gowda, B. T., Foro, S., Suchetan, P. A. & Fuess, H. (2010). *Acta Cryst.* E**66**, o326. 10.1107/S1600536809055482 PMC2979754 21579756

[bb2] Oxford Diffraction (2009). *CrysAlis CCD* and *CrysAlis RED* Oxford Diffraction Ltd, Yarnton, England.

[bb3] Sheldrick, G. M. (2008). *Acta Cryst.* A**64**, 112–122. 10.1107/S0108767307043930 18156677

[bb4] Spek, A. L. (2009). *Acta Cryst.* D**65**, 148–155. 10.1107/S090744490804362X PMC2631630 19171970

[bb5] Suchetan, P. A., Gowda, B. T., Foro, S. & Fuess, H. (2010*a*). *Acta Cryst.* E**66**, o766. 10.1107/S160053681000783X PMC2983762 21580610

[bb6] Suchetan, P. A., Gowda, B. T., Foro, S. & Fuess, H. (2010*b*). *Acta Cryst.* E**66**, o1040. 10.1107/S1600536810012808 PMC2979121 21579101

[bb7] Suchetan, P. A., Gowda, B. T., Foro, S. & Fuess, H. (2010*c*). *Acta Cryst.* E**66**, o1253. 10.1107/S160053681001559X PMC2979647 21579357

